# Humeral Head Replacement and Reverse Shoulder Arthroplasty for the Treatment of Proximal Humerus Fracturesm 

**DOI:** 10.2174/1874325001711011108

**Published:** 2017-09-30

**Authors:** Aaron Andrew Frombach, Kendra Brett, Peter Lapner

**Affiliations:** Division of Orthopaedics, Ottawa Hospital Research Institute, University of Ottawa, Ontario, Canada

**Keywords:** Humeral head replacement, Proximal humerus fracture, Proximal humerus fracture-dislocation, Proximal humerus hemiarthroplasty, Reverse shoulder arthroplasty, Tuberosity

## Abstract

Acute proximal humeral fractures in the elderly are generally treated non-operatively if alignment is acceptable and in stable fracture patterns. When operative treatment is indicated, surgical fixation is often difficult or impossible to obtain. Hemiarthroplasty has long been the standard of care. However, with its reliance on tuberosity healing, functional outcomes and patient satisfaction are often poor. Reverse shoulder arthroplasty has emerged as a new technology for treating proximal humeral fractures but the indications for its use remain uncertain. While not conclusive, the evidence suggests that reverse shoulder arthroplasty yields more consistent results, with improved forward elevation and higher functional outcome scores. The primary advantages of hemiarthroplasty are improved shoulder rotation and shorter operative time. Complication rates do not vary significantly between the two options. Although higher quality trials are needed to further define the role of reverse shoulder arthroplasty, current evidence suggests that this is a reasonable option for surgeons who are highly familiar with its use.

## 1 INTRODUCTION

Acute fractures of the proximal humerus are the third most common type of fracture in the elderly [1]. Depending on patient and fracture characteristics, these can often be effectively treated non-operatively, as is the case in 80-85% of patients [2]. When surgery is indicated, options include shoulder arthroplasty, or reconstructive options including fixation with proximal humeral locked plate constructs in the physiologically young with adequate bone stock [3]. When arthroplasty is indicated, humeral head replacement arthroplasty (HHR) has historically been the treatment of choice for proximal humeral fractures as advocated initially by Neer [2]. However, in recent years there has been a trend towards reverse total shoulder arthroplasty (RSA). This has occurred in part due to the prevalence of rotator cuff deficiency in the affected patient population. In addition, non-union or malunion of the tuberosities have been associated with poor results with hemiarthroplasty; complete healing does appear to be necessary for a successful outcome following RSA [4]. Finally, RSA may allow for a shorter period of immobilization post-operatively given its semi-constrained design.

## 2 BASIC SCIENCE

### 2.1 Epidemiology

Fractures of the proximal humerus are very common in the elderly population, resulting in approximately 6% of all fractures in adults [1]. While up to 85% are minimally displaced and can be treated non-operatively, this still leaves a significant number of fractures that require operative treatment.

### 2.2 Diagnosis/Classification

In 1970, Neer described the classification that is most prevalent today [2]. This classification system is based on the number of main anatomical fragments, or parts (two, three, or four) which are defined as the humeral head, greater tuberosity, lesser tuberosity and humeral shaft, having displacement of 1cm or angulation of 45 degrees from their anatomic position. Variant patterns include head splitting fractures which are generally treated with arthroplasty except in the physiologically young and active patients, fracture dislocations which have a high incidence of associated neurovascular injury, and fractures of the anatomic neck with minimal calcar remaining that are prone to avascular necrosis of the humeral head [3].

## 3 CLINICAL DECISION MAKING

High demand and physiologically young patients may have increased tolerance for repeat surgery and it may be reasonable to attempt a reconstruction even in higher grade proximal humerus fractures in this population. Low functional demand patients may be better served with a replacement, and those with pre-morbid symptoms of rotator cuff pathology or evidence of rotator cuff arthrosis may be better served with RSA [4]. RSA is contraindicated in the setting of axillary nerve injury however.

Based on radiographic criteria, primary arthroplasty may be indicated if healing is unlikely or if there is vascular compromise of the humeral head [5]. Fractures of articular surface (head splitting fractures) are considered a strong indication for primary arthroplasty. Factors associated with vascular compromise of the humeral head constitute relative indications for primary arthroplasty and include the following: anatomic neck fractures with minimal blood supply remaining for the humeral head (calcar spike < 8mm remaining attached to the humeral head), lack of a medial hinge and gross angulation and/or displacement of the fragments, specifically the humeral head and/or the tuberosities [5]. Radiographs are often sufficient for preoperative planning but a CT may be obtained to confirm a head splitting fracture and to further delineate the extent of articular surface involvement, the degree of comminution, and the relative positions of the fragments.

## 4 OPERATIVE TECHNIQUES

Both HHR and RSA have been extensively described in the literature and the operative techniques are not within the scope of this article [6]. With rare exception, both techniques utilize a deltopectoral approach to the shoulder with various options for subscapularis management. In hemiarthroplasty the component stem is generally inserted with cemented technique and the prosthetic humeral head articulates with the native glenoid. If the fracture involves the tuberosities, they are fixated to the shaft and bone graft may be used to augment healing potential. With fracture treatment, RSA fixation is generally with cement although cementless options exist.

## 5 HUMERAL HEAD REPLACEMENT – CLINICAL OUTCOMES

In one systematic review of patients treated with HHR for proximal humeral fractures, 41% of patients reported unsatisfactory outcomes [7]. However 40% also reported satisfactory to excellent outcomes, which included pain only with vigorous activity. Overall, with an analysis of over 800 HHR cases, the mean forward elevation was 106 degrees, external rotation 30 degrees and the Constant score was 56.6. Eleven percent of patients had complications related to tuberosity fixation and 6.8% had superior subluxation of the humeral head on follow-up radiographs [7].

## 6 REVERSE SHOULDER ARTHROPLASTY *VS.* HUMERAL HEAD REPLACEMENT

No long term outcome studies have been published on RSA in the fracture setting. This has led many authors to question its durability and the wisdom of using this implant in physiologically younger patients for fracture [4]. Current indications for RSA include advanced age and decreased functional demand, rotator cuff arthropathy, chronic fractures, or failed hemiarthroplasty [8]. Risks specific to RSA include glenoid component loosening, glenoid notching, infection, complex regional pain syndrome, and proximal bone resorption. Intraoperative fracture of the glenoid may prevent insertion of the glenoid baseplate [8]. In systematic reviews comparing RSA with HHR, both Ferrell *et al.* and Namdari *et al.* found increased complication rates with RSA as compared to HHR, while Mata-Fink *et al.* reported better outcomes overall with RSA compared to HHR based on the Constant score, ASES and OSS [9-11].

Range of motion (ROM) comparison between the two procedures by Mata-Fink *et al.* demonstrated superior forward elevation with RSA with mildly decreased external rotation [11]. Ferrell *et al.* similarly found superior forward elevation without a significant decrease in external rotation with RSA [9]. However, Namdari *et al.* did not observe any significant range of motion differences between the two prostheses [10], and with follow-up averaging under 30 months, the Constant and ASES scores were not significantly different between HHR and RSA in their analysis. Complication rates were higher with RSA but revision rates were higher with HHR in the analysis by both Ferrell *et al.* and Namdari *et al.* [9, 10]. Mata-Fink *et al.* did not find a significant difference in either revision or complication rates [11]. It is important to consider however that a revision option exists for HHR (to RSA) which may explain why this option may be more readily considered than revision of a RSA in which reconstructive options are far more limited.

The results of these three systematic reviews did not demonstrate clear superiority of one prosthetic option over the other. Both appear to be viable options; further prospective studies are needed to further elicit differences in functional outcomes and to further define optimal indications.

Sebastia-Forcada *et al.* conducted the only randomized trial in the literature to date comparing RSA with HHR for acute proximal humeral fractures [12], Table **1**. All patients underwent CT scan imaging. There was a minimum 2 year follow-up. A single modular system was used and the post-operative rehabilitation program was standardized across both groups. Functional outcome measures including the Constant, DASH, UCLA scores, active range of motion and tuberosity healing, were significantly higher in patients treated with RSA. The revision rate was lower with RSA. Functional outcomes were poorer with revision of HHR to RSA compared to cases treated with RSA primarily. Successful outcomes in the HHR group were dependent on tuberosity healing. The presence of an irreparable rotator cuff was a strong predictor of failure in HHR [12].

Baudi *et al.* [13] reported greater improvement in the Constant, ASES score and ROM in patients treated with RSA over hemiarthroplasty. Sub-group analysis demonstrated that this difference was most pronounced in the most elderly patient group (>75 years). No significant differences were observed in the DASH scores between groups however [13].

Cuff and Pupello [14] compared HHR and RSA in a prospective study. Fifty-three patients with three and four part fractures in patients older than 70 years were included. ASES, SST scores, ROM, and patient satisfaction were significantly higher in RSA than in HHR. Functional outcome measures and ROM were significantly higher in patients with healed tuberosities, although these scores were lower than in patients treated with RSA regardless of tuberosity healing [14].

In another retrospective study that compared HHR with RSA, Gallinet *et al.* [15] observed that 21 patients treated with HHR had worse Constant scores and decreased forward elevation and abduction than the 19 patients treated with RSA. However, the DASH scores were not significantly different and the RSA group had increased rotation. Radiographic complications included three patients with tuberosity malunion or non-union in the HHR group compared with 15 cases of scapular notching in the RSA group. However, the clinical relevance of the notching was not discussed; patients with malunited or non-united tuberosities in the HHR group had significanty worse functional outcomes [15].

In 2013, Boyle *et al.* [16] reported their findings from the New Zealand Joint registry. This is currently the largest study in the literature, with 368 patients skewed heavily to HHR (313 *vs* 58 RSA). The Oxford Shoulder Score was higher in the RSA group than the HHR group at 5 years. Revision rates were not significantly different between groups [16].

In a much smaller study, Young *et al.* [17] did not observe any significant differences in functional scores (ASES, Oxford), ROM or complications rate between 10 RSA and 10 matched HHR patients.

Garrigues *et al.* [18]. reported on 19 patients with proximal humerus fractures Fig. (**1**) treated with HHR or RSA with followup averaging 3.6 years. Patients with RSA had significantly better functional outcomes scores and satisfaction. The RSA patients were older, with a mean age of 80 years compared to 69 years in the HA group; RSA patients had better forward elevation, and higher functional scores as measured by ASES, University of Pennsylvania score and Single Assessment Numeric Evaluation. Quality of life measures and rotation were not significantly different between groups [18].

In a cost-effectiveness analysis, Chalmers *et al.* [19] observed that RSA was less expensive overall when total cost including all factors including post-operative rehabilitation were considered.

## CONCLUSION

In summary, both HHR and RSA appear to offer good pain relief with no difference in DASH scores, a measure of disability in daily life, in studies that used this outcome measure. However, functional outcomes in HHR are significantly lower when the tuberosities do not heal, a factor which does not appear to affect the functional outcomes in RSA with the exception of rotation. Survivorship continues to remain a concern with RSA, although revision rates appear to be higher with HHR. The cost of hemiarthroplasty prosthesis is considerably lower than RSA implants; however data suggests that HHR is more expensive when the higher rehabilitation costs are considered. While both HHR and RSA are reasonable implant choices for elderly patients with acute proximal humerus fractures, RSA appears to carry certain advantages, particularly in elderly and low-demand patients, because a successful outcome is much less contingent on tuberosity healing.

## Figures and Tables

**Fig. (1):**
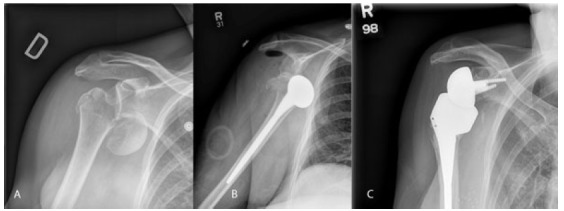
**A**) plain xrays of comminuted acute proximal humerus fracture-dislocation. Note comminution of tuberosities and humeral head displacement with minimal calcar remaining. **B**) AP of post-operative day one humeral head replacement showing dislocation of prosthesis from glenoid. **C**) Six month post-operative follow-up AP imaging following revision to reverse total shoulder arthroplasty. Patient was doing functionally well with no complaints of pain and range of motion continuing to improve.

**Table 1:** Summary of studies comparing humeral head replacement and reverse shoulder arthroplasty.

**Study**	**Population (n, mean age, % male)**	**Comparison**	**Fracture Type**	**Follow Up (Months, Outcome Measures)**	**Main Findings**	**Limitations**	**Conclusion**
Gallinet 2009	HHR n=17 74 (49-95) 12% male RSA n=16 74 (58-84) 19% male	Short term Retrospective Non-randomized HHR done 1996-2001 RSA done 2002-2004	Three or four part displaced fractures	HHR 16.5mth (6-55) RSA 12.4mth (6-18) Active joint amplitude Constant DASH Standard X-ray	RSA had better: -anterior elevation (97° vs. 54°)* -Abduction (91° vs. 60°)* -Constant (53 vs. 39)* HHR had better: -external rotation (14° vs. 9°) No difference in DASH 3 abnormal tuberosity fixations in HHR 15 glenoid notches in RSA	Retrospective Non-randomized HHR vs. RSA was determined by year of Sx Small n Short follow up	While clinical results were better for RSA, patient did not necessarily experience a benefit in quality of life (DASH) RSA only used in patients >70years.
Young 2010	HHR n=10 75.5 20% male RSA n=10 77.2 0% male	Retrospective Non-randomized HHR done from 2003-2005 RSA done from 2005-?	Three and four part factures	HHR 44mth (24-56) RSA 22mth (16-37) Satisfaction ASES Oxford shoulder score X-rays	No differences in outcome scores between groups Two complications in HHR	Non-randomized Small n HHR vs. RSA was determined by year of Sx Different follow up periods	The anticipated functional gains of RSA were not realized Larger prospective trials are needed.
Cuff 2013	HHR n= 23 74.1 (70-88) 39% male RSA n=24 74.8 (70-86) 42% male	prospective non-randomized 26 HHR pt. then 27 RSA pt	4 Part, or 3 part w/ severe comminution of greater tuberosity, or split of humeral head	30mth (24-48mth) ASES SST Satisfactory or not ROM Standard X-rays	RSA had better: -ASES (77 vs. 62)* -SST (7.4 vs. 5.8)* -forward elevation (139° vs. 100°)* -satisfaction (91% vs. 61%)* -tuberosity healing (83% vs. 61%) (NS) Similar complication rates HHR success was dependent on tuberosity healing	Non-randomized HHR had longer follow up No pre-op ASES or SST	RSA had better clinical outcomes than HA HHR outcomes depended on tuberosity healing, RSA did not.
Baudi 2014	HHR n=28 71.4 RSA N=25 77.3 7 male 46 female	Retrospective Non-randomized Examined 3 ways: -whole group - >65 <75 ->75	Four part displacement fractures	27.5mth (12-64 mth) Constant ASES DASH Strength in abduction, ER1, ER2 Standard X-rays	Whole group RSA had better: -Constant (56 vs. 42)* - ASES (69 vs. 51)* -forward elevation (131° vs. 89°)* -abduction (128° vs. 82°)* -tuberosity consolidation (84% vs. 37%)* >65 <75 RSA had better: -Constant (61 vs. 37)* - forward elevation (135° vs. 77°) * -abduction (136° vs. 67°)* >75 RSA had better -Constant (52 vs. 40)* -ASES (67 vs. 46)* - forward elevation (125° vs. 35°)* -abduction (120° vs. 80°)* No differences in DASH	Retrospective, non-randomized 3 types of prosthesis used Multiple comparisons and analyses performed No group characteristics Follow up is unclear	Authors fail to make any definitive conclusion.
Boyle 2013	55 RSA (79.6 yrs, 7%) 313 HHR (71.9 yrs, 22%)	Retrospective Registry Study	Acute proximal humerus fracture	Oxford Shoulder Score Mortality Revision	OSS Same at 6/12 OSS at 5 years RSA=41.5 OSS at 5 years HHR=32.3 Revision and mortality same	No radiographic evaluation No preop characteristics Unequal demographics	RSA had higher functional scores at 5 year follow-up than HHR.
Chalmers 2014	HHR=9 (age=72 Male= 25) RSA=9 (age=77 Male= 22)	Retrospective Case-Controlled Cohort 9 RSA 9 HHR 9 ORIF	Three and Four part displaced fractures from ground level fall	SF12 SST ASES ROM (AFE) Cost analysis	No difference in ASES, SST or SF12 Faster and more predictable return of AFE RSA equal cost to ORIF, cheaper than HHR by ~$5000/patient	Small, retrospective cohort study with short F/U No pre-op characteristics	Significantly less expensive with better outcomes of RSA than HHR
Garrigues 2012	11 RSA 12 HHR	Retrospective Review	3 and 4 part fractures	F/U=3.6 years ASES, AFE, UPenn Shoulder Score, SANE			
Sebastia-Forcada 2014	HHR n=30 73.3 (70-83) 17% male RSA n=31 74.7 (70-85) 13% male	Prospective RCT Blinded HHR vs. RSA	Displaced 4 part factures, fracture dislocations with 3 part fractures, head splitting with more than 40% articular surface involvement	HHR 27.7 mth (24-49) RSA 29.4 mth (24-44) Constant UCLA QuickDASH Standard X-rays	RSA had better: -functional scores and active ROM (but not dif in internal rotation) -Constant (56 vs. 40)* -Pain [mild or non (14) vs. moderate or severe (8.8)]* -Anterior forward (120 vs. 79)* -UCLA score (29 vs. 21)* -DASH (17 vs. 24)* -rate of clinical failure (26% vs. 57%)*	Low healing rate of tuberosities Short follow up limits interpretation of results.	Significantly better functional outcome and revision rate were obtained with RSA Worst outcomes of HHR with failure of tuberosities to heal; but healing of tuberosities was not associated with RSA success

## LIST OF ABBREVIATIONS

HHR: = Humeral Head Replacement
RSA: = Reverse Shoulder Arthroplasty
